# Supplementary material for: Splice-Junction-Based Mapping of Alternative Isoforms in the Human Proteome
Source: Cell Rep. Author manuscript; Available in PMC 2020 Jan 15. (PMC6961840; doi:10.1016/j.celrep.2019.11.026)

A

sp|Q96AT9|RPE_HUMAN|ENSG00000197713|SE1|25131|chr2|210017559|210018221|+2|r4|T4
SSILNSDLANLGAECLR q value: 0.00039754 Tr_novel:TRUE RefSeq_Novel:TRUE
Search result spec prec mz: 611.6439 Actual spec prec mz: 611.64386
Fragments matched per AA: 2.65 Proportion of top 20 peaks matched: 0.2

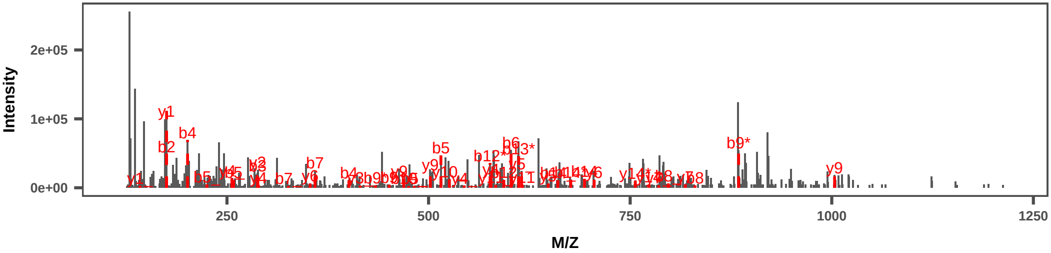

B

Scatterplot of predicted elution time
Fitting R2: 0.849
Novel peptide residual Z score: −1.88
Number of peptides: 2550

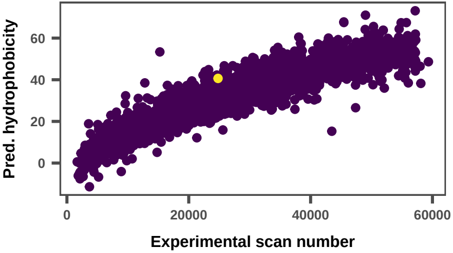

C

Distributions of residuals from best−fit line
of predicted RT vs Expt. scan number
Line: Z score of novel peptide
Z: −1.88

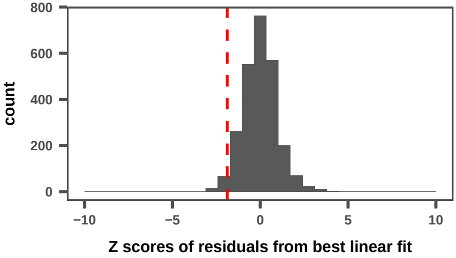

Supplement: 2 [file NIHMS1546469-supplement-2.zip › DF1/PXD010154/Heart/Heart_61_RPE_SSILNSDLANLGAECLR.pdf]
